# pH-Dependent Deformations of the Energy Landscape of Avidin-like Proteins Investigated by Single Molecule Force Spectroscopy

**DOI:** 10.3390/molecules190812531

**Published:** 2014-08-18

**Authors:** Melanie Köhler, Andreas Karner, Michael Leitner, Vesa P. Hytönen, Markku Kulomaa, Peter Hinterdorfer, Andreas Ebner

**Affiliations:** 1 Institute of Biophysics, Johannes Kepler University Linz, Gruberstrasse 40, 4020 Linz, Austria; E-Mails: melanie.koehler@jku.at (M.K.); peter.hinterdorfer@jku.at (P.H.); 2 Center for Advanced Bioanalysis, Gruberstrasse 40, 4020 Linz, Austria; E-Mails: andreas.karner@cbl.at (A.K.); michael.leitner@cbl.at (M.L.); 3 Institute of Biomedical Technology, University of Tampere, FI-33014 Tampere, Finland; E-Mails: vesa.hytonen@uta.fi (V.P.H.), markku.kulomaa@uta.fi (M.K.); 4 Fimlab Laboratories Ltd., FI-33101 Tampere, Finland

**Keywords:** avidin mutant, avidin-biotin, force spectroscopy, molecular recognition, pH dependence, single molecules, biophysics

## Abstract

Avidin and avidin-like proteins are widely used in numerous techniques since the avidin-biotin interaction is known to be very robust and reliable. Within this study, we investigated this bond at the molecular level under harsh conditions ranging from very low to very high pH values. We compared avidin with streptavidin and a recently developed avidin-based mutant, chimeric avidin. To gain insights of the energy landscape of these interactions we used a single molecule approach and performed the Single Molecule Force Spectroscopy atomic force microscopy technique. There, the ligand (biotin) is covalently coupled to a sharp AFM tip via a distensible hetero-bi-functional crosslinker, whereas the receptor of interest is immobilized on the probe surface. Receptor-ligand complexes are formed and ruptured by repeatedly approaching and withdrawing the tip from the surface. Varying both pulling velocity and pH value, we could determine changes of the energy landscape of the complexes. Our results clearly demonstrate that avidin, streptavidin and chimeric avidin are stable over a wide pH range although we could identify differences at the outer pH range. Taking this into account, they can be used in a broad range of applications, like surface sensors at extreme pH values.

## 1. Introduction

Avidin and avidin-like proteins ranging from avidin and streptavidin to more recently developed mutants like chimeric avidin and traptavidin [1,2,3] are known to play a major role in life-science applications (for reviews see [4,5,6]). Avidin-like proteins are commonly built up as a homotetramer, whereby each monomer has a single eight-stranded β-barrel acting as binding pocket for D-biotin. The wide range of biochemical, biophysical, pharmaceutical, and medical applications is a logical result of numerous advantages: (i) avidin (and most of the avidin-like proteins) shows an extremely high affinity towards the water-soluble vitamin D-biotin (K_d_ ~10^−15^ M). D-Biotin itself is a very small molecule that can be easily bound covalently to proteins with a low probability to harm their functional properties. (ii) The system is available for a broad range of applications and comparable cheap. In addition (iii) avidin/biotin is known to be very robust and reliable. In previous studies the capability of (strept)avidin (mutants) to bind biotin after pretreatment at different environmental conditions ranging from detergents [7] and organic solvents, temperature changes [2,8], up to harsh pH conditions [2] was investigated. In all these studies an extraordinary stability of these proteins could be demonstrated. Within this study we explored the biotin binding behavior at extreme conditions of three proteins: avidin, streptavidin and the avidin mutant chimeric avidin. Chimeric avidin is an avidin based mutant known to have an increased thermal and pH stability and a better resistance against the proteolytic activity of proteinase K. Chimeric avidin is prepared by replacing a segment in avidin with corresponding sequence stretch from avidin related protein 4. In addition, the isoleucine at position 117 is exchanged by tyrosine, most probably resulting in a stabilization effect due to π-π interaction of two tyrosines from adjacent subunits. In a recent study [1] the biotin binding activity of these proteins after exposure of harsh conditions like pH values ranging from 1 to 13 was measured by using enzyme-linked biotin as a probe. In contrast to the previous study where the protein activity was measured at near physiological pH conditions–just after the harsh environmental treatment—we now investigated the ability of avidin/streptavidin/chimeric avidin to bind exactly at extreme pH values. This allows getting insights into potential applications at harsh conditions. 

Biotin is known to dissociate from avidin and streptavidin overcoming three or two energy barriers [9,10]. The highest barrier there acts limiting for the dissociation kinetics whereas the lower barriers can be seen as semi-stable intermediate states, stabilized by newly formed weak bonds while dissociation. Although numerous techniques like surface plasmon resonance or quartz crystal microbalance are available to determine the averaged unbinding behavior of biotin from immobilized avidins, the exploration of the energy landscape requires a single molecule technique. Beside the surface force apparatus [11,12], optical or magnetic tweezers [13], and bio-membrane force probe [14], single molecule force spectroscopy (SMFS) is optimally suited for this purpose. (Strept)avidin–biotin was one of the first receptor–ligand systems investigated with this technique [15,16,17,18] and is still in the focus of recent research. In earlier studies changes on this energy landscape introduced by protein mutations [19,20,21] and by varying environmental conditions [22,23] were investigated. Furthermore effects of multiple biotin binding [24,25], improvements in measurement [26] and interpretation [27] and simulations of the system were published [28,29,30]. SMFS allows measuring the rupture forces under different force loads, and, according to Evans theory, enables visualization of possibly existing different energy barriers yielding in the parameters of the energy potential (*i.e.*, k_off_ and x_β_). Within this study we applied AFM imaging and force spectroscopy to reveal possible changes of the energy landscape of these proteins in the presence of strongly different H^+^ concentrations ranging from pH 1 to 12.75.

## 2. Results and Discussion

### 2.1. Tip and Support Functionalization

**Figure 1:**
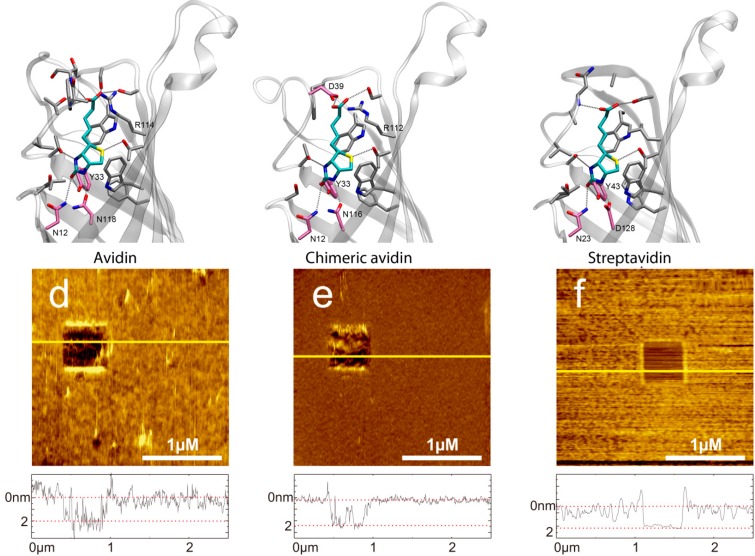
(**a**) Molecular structure of D-biotin in the β-barrel avidin (PDB 2AVI). (**d**) AFM image of a covalently bound avidin layer. Scratching of a 0.5x0.5 µm area results in a hole. In the cross-section below (corresponding to the yellow line in the AFM image) the height difference of 2.07 ± 0.47 nm can be seen between avidin and the support. (**b**) Molecular structure of D-biotin in the β-barrel chimeric avidin prepared by positioning the ligand to the apo structure of chimeric avidin (PDB 3MMO) by using avidin-biotin complex as a template. (**e**) AFM image of a covalently bound chimeric avidin layer. Image size is 2.5 × 2.5 µm, z-scale bar is 7 nm. The height difference in the cross-sections is 1.86 ± 0.26 nm. (**c**) Molecular structure of D-biotin in the β-barrel streptavidin (PDB 1MK5). (**f**) AFM image of a covalently bound streptavidin layer. Image size is 2.5 × 2.5 µm, x-scale bar is 1 µm. The height difference in the cross-sections is 2.03 ± 0.15 nm. The molecular representations were prepared by using the program VMD.

**Figure 2:**
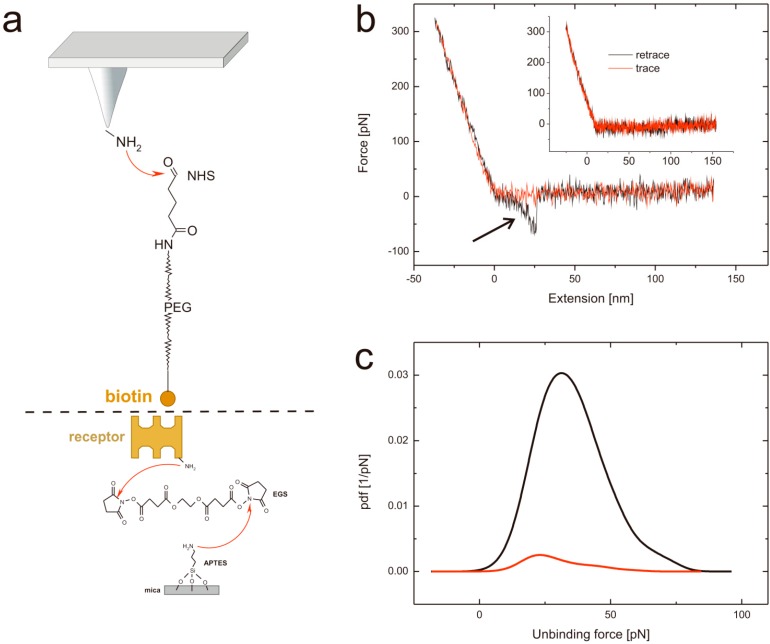
(**a**) Tip and support chemistry. Upper part: APTES functionalized AFM tips are reacted with the heterobifunctional PEG tether NHS-PEG-biotin resulting in a covalent amide bond formation. Lower part: avidin (or avidin-like protein) is covalently bound to APTES coated mica using a homo-bifunctional EGS crosslinker. (**b**) Typical force distance cycle. The distance dependent cantilever bending is shown in red for the approaching period and in black for the retraction. In the latter a typical unbinding event, visible as parabolic shaped downwards bending can be seen (highlighted with an arrow). In contrast, the inset represents a force distance cycle without any specific interaction. (**c**) Specificity proof exemplary shown for streptavidin at a pulling velocity of 400 nm/s at pH 7. The black line represents the probability density function using a biotin tethered tip, whereas the red line shows the probability density function of the very same system after blocking the tip by adding free streptavidin.

Performing single molecule force spectroscopy experiments require both stable tethering of the ligand to the tip as well as stable coupling of the corresponding ligand to the solid support. To ensure tight binding covalent chemistry is best suited. Thus, we silanized mica using the APTES gas phase protocol [31] resulting in reactive amino residues. A short homo bi-functional amino-reactive linker was used to ligate avidin proteins to the surface via amino groups. In Figure 1 the amino-acids in the β-barrel responsible for biotin binding of (a) avidin, (b) chimeric avidin, and (c) streptavidin are shown. In all cases the covalent coupling is performed by using lysine residues on the outer protein surface. To test the binding protocol, the protein functionalized surfaces were imaged using constant force contact mode imaging with a soft cantilever to avoid denaturation due to too high indentation forces. After imaging an area of 2.5 × 2.5 µm the scan size was reduced to 0.5 × 0.5 µm and the indentation force was increased significantly to remove the proteins. As a result, the height differences between the protein surface height level and those of the scratched areas got visible. As shown in Figure 1 (lower part) the height of (d) the avidin layer was 2.07 ± 0.47 nm, of (e) chimeric avidin 2.03 ± 0.15 nm, and that of (f) streptavidin 1.86 ± 0.26 nm. The observed heights were somewhat lower compared to those expected by the 3D structures, which can be explained by the applied indentation force of the AFM tip resulting in a compression of the proteins. Although minor differences especially in the surface roughness are evident it is proven that the coupling yielded in a dense protein layer usable for further SMFS measurements. For tethering biotin to the outer tip apex a well-established protocol as depicted in Figure 2a based on APTES gas phase silanization of silicon nitride tips followed by covalent binding of the hetero bi-functional poly(ethyleneglycol) crosslinker NHS-PEG(18)-biotin [32] was used.

### 2.2. Force Distance Cycles, Data Evaluation and Specificity Proof Experiments

To measure the interaction forces between biotin and avidin force distance cycles (FDCs) were performed (Figure 2b). The biotin-functionalized tip was approached to the surface (red line) without observable bending of the cantilever. From the moment of contact, further approaching resulted in an upwards bending of the cantilever which was stopped at a previously set force limit of typically 200–400 pN.

In the retraction period (black line) the cantilever bending gets reduced and, in the moment of losing contact, finds its resting position again. In the whole period of contact biotin was able to form a complex with avidin, streptavidin or chimeric avidin. In case of complex formation a second – this time downwards – bending appears as highlighted by an arrow in Figure 2b. The non-linear increase of force while withdrawing the cantilever with a constant pulling velocity is a result of the stretching of the PEG tether, according to the worm-like-chain model. Most important the rupture (unbinding) force (*i.e.*, the difference between the maximum downward bending and the resting position) can be determined directly by translating the bending according to Hook’s law into a force. In case of no complex formation (as shown in Figure 2b inset) this typical second bending event does not appear. The unbinding force differs slightly at the same settings as a result of the thermal energy contribution. Thus, sufficient statistics are necessary, which is realized by repeating the experiment at least 1,000 times.

By plotting the probability density function (PDF) [33] which can be seen as error weighted histogram of the rupture forces, the most probable unbinding event can be determined. In Figure 2c black line a PDF constructed from FDCs of streptavidin and biotin measurements (at a pulling velocity of 400 nm/s and at pH 7) is shown with a most probable rupture force at 31.3 pN.

Although the shapes of the unbinding events in the FDCs give a clear hint that the rupture is caused by pulling on the PEG tethered ligand from the surface, a specificity proof is needed to exclude nonspecific binding of the ligand to the surface. For this, we repeated the experiment with the very same tip and surface but performed a tip block by incubation of the tip in a solution of “free” streptavidin before the experiments. As a result the binding probability dropped down from 11.2% before the block (Figure 2c, black line) to 1.4% after the block (Figure 2c, red line). PDFs are set in relation to their binding probability. The same proof experiment was performed for avidin (data not shown) resulting in 15.5% before and 1.2% after the block and for chimeric avidin (data not shown) yielding in a decrease from 13% to 1.7%. Thus, it can be guaranteed that all measured interaction forces correspond to highly specific interactions.

### 2.3. Influence of the pH Value on the Binding Probability

**Figure 3:**
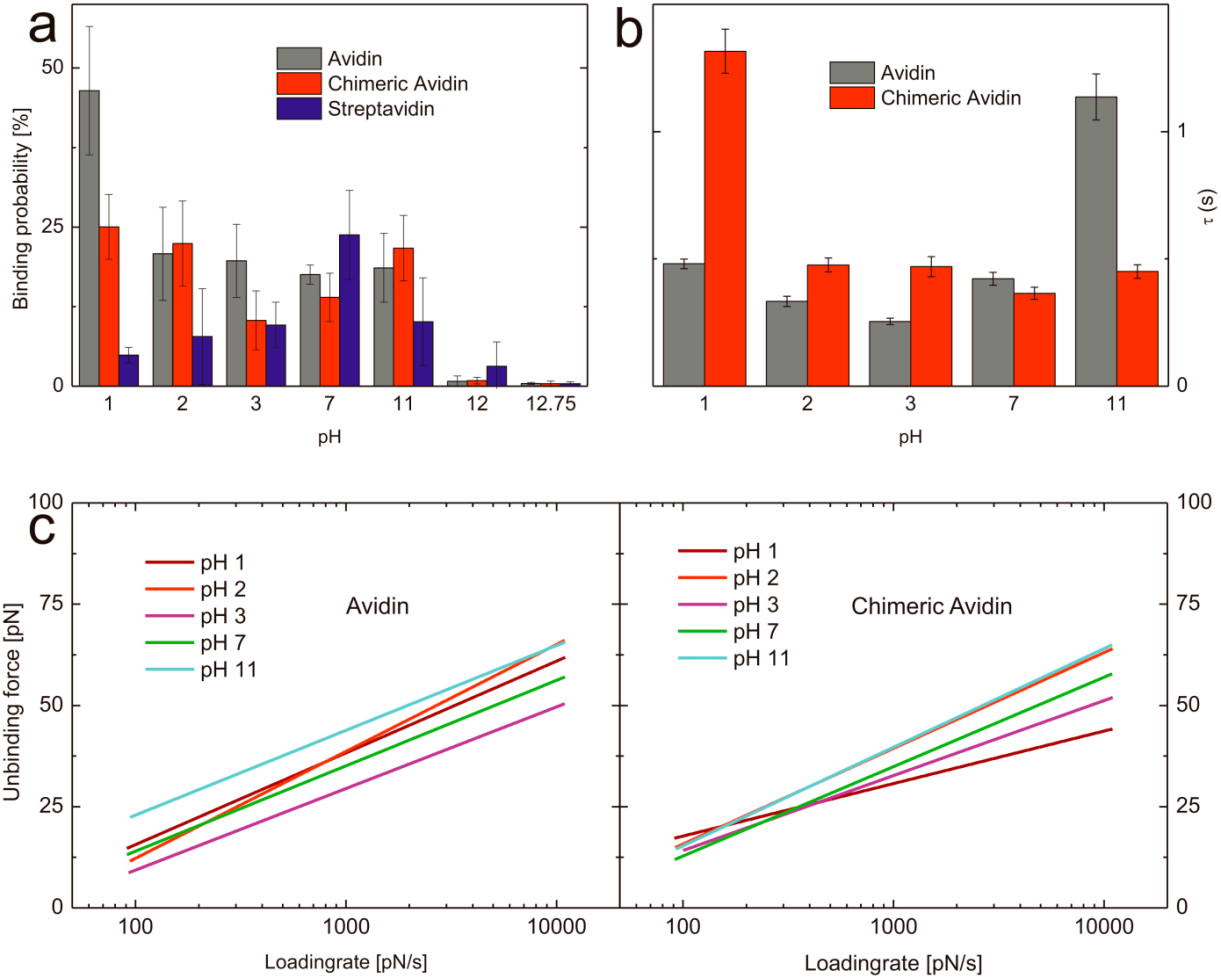
(**a**) Comparison of the binding probabilities of D-biotin with avidin (gray), chimeric avidin (red), and streptavidin (blue) at different pH values. (**b**) Complex lifetimes τ of avidin-biotin (gray) and chimeric avidin-biotin (red) at different pH values. (**c**) Loading rate dependence of the most probable unbinding force of avidin (left) and chimeric avidin (right) at pH values ranging from 1–11.

In order to determine the influence regarding the pH on the binding probability of biotin with avidin/streptavidin/chimeric avidin we performed SMFS experiments at different pH values. To have comparable results all data sets were acquired with the very same tip and also by using the same proteins immobilized on a solid surface. In all cases the measurements were done beginning with neutral pH and ended with the harshest conditions. To prove that the activity of the avidin like proteins was not affected by the previous conditions (pH treatment) we performed FDCs at harsh pH conditions and afterwards exchanged the buffer back to neutral and again determined the binding behavior towards biotin. For all proteins it was shown that even after exposure (e.g., pH 12.75, where no stable complex formation could be observed for all proteins) the activity was recovered completely after changing back the pH value to seven. The mean binding probability at pH 7 was 17 ± 7.5% (n = 3) which dropped after changing the buffer at pH 12.75 to 0.4 ± 0.25% (n = 3), whereas by changing back to a pH 7 buffer the binding ability recovered again nearly to the same value as before (13.8 ± 1.30%). Thus we can conclude, that no denaturation was observable. To allow comparable results all mentioned binding probabilities (if not stated otherwise) are acquired at pulling velocities ranging from 50–300 nm/s (*i.e.*, 50, 100, 200, 300 nm/s). To ensure that the recovered probability is not caused by unspecific interaction, we performed specificity proof measurements by blocking the biotin on the tip. As a result the binding probability dropped down to 1.4 ± 0.13% (n = 3) and thus successfully verified the high specificity of the interaction. As to determine the binding probability over a broad pH range we performed single molecule force spectroscopy at pH values ranging from 1 to 12.75. Since we did not expect a lowering in binding performance at gently conditions we only measured pH 7 as a reference and focused on pH values of 3 and lower as well as of 11 and higher (Figure 3 and Table 1). The ability of avidin to bind biotin was very comparable over the range from pH 2 to pH 11. At lower H^+^ concentrations the probability dropped down significantly. In contrast, the binding probability of biotin towards avidin significantly increased at pH 1 (46.43 ± 7.58%, n = 4). It can be excluded, that this is only a result of charge driven increased adhesion since the specificity proof by blocking the tip tethered biotin resulted in a clear decrease of the binding probability (5.4%, n = 1) and thus verified the specificity.

**Table 1:** Kinetic off-rates (k_off_) and width of energy barriers (x_β_) for avidin and chimeric avidin at a loading rate region ranging from 100–10,000 pN/s.

	Avidink_off_ [s-1]	x_β_ [Å]	Chimeric avidink_off_ [s-1]	x_β_ [Å]
pH 1	2.08 ± 0.08	4.18 ± 0.04	0.76 ± 0,05	7.31 ± 0.08
pH 2	3.00 ± 0.18	3.59 ± 0.07	2.10 ± 0.12	4.01 ± 0.06
pH 3	3.93 ± 0.19	4.70 ± 0.07	2.13 ± 0. 18	5.12 ± 0.10
pH 7	2.37 ± 0.14	4.49 ± 0.07	2.74 ± 0.18	4.29 ± 0.08
pH 11	0.88 ± 0.07	4.52 ± 0.08	2.22 ± 0.13	3.89 ± 0.06

One possible explanation for this observation could be a change in the conformation of the loop L3,4 in avidin. This loop acts as a gatekeeper in avidin, and is probably heavily responsible for the very slow dissociation of biotin from avidin. Although apo-avidin structures indicate flexible conformation of the loop [34,35], it is possible, that L3,4 has significant negative impact on the association velocity. Therefore, our data suggest that avidin exhibits a more open conformation at low pH, which, together with charge-mediated effects, facilitates rapid biotin binding. Very high pH values resulted in a complete abolishment of the binding (pH 12: 0.75 ± 0.84%, n = 4; pH 12.75: 0.43 ± 0.15%, n = 4). Chimeric avidin shows comparable results at pH 7 (12.68 ± 2.40%, n = 4) and 11 (19.83 ± 5.5%, n = 4), whereas at low pH values the probability first slightly decreases at pH 3 (10.33 ± 5.39%, n = 4) but increases again already at pH 2 (22.00 ± 7.83%, n = 4) and has a significantly higher value at pH 1 (25.68 ± 4.84%, n = 4) compared to pH 7. In contrast, the binding probability of streptavidin towards biotin shows other pH dependent behavior. At neutral pH streptavidin shows the highest binding probability of 23.78 ± 6.98% (n = 4) towards biotin whereas, lowering and increasing of the pH resulted in a diminishment of the probability of complex formation. Both, the binding probability at pH 11 (10.13 ± 6.88%, n = 4) as well as at pH 3 (9.60 ± 3.60%, n = 4) was significantly lowered. pH 2 (7.80 ± 7.53%, n = 4) and pH 1 (4.88 ± 1.22%, n = 4) resulted in even lower values for the probability of streptavidin-biotin complex formation but still higher than pH 12 (3.13 ± 3.83%, n = 4) and pH 12.75 (0.38 ± 0.33%, n = 4).

Summed up, we could demonstrate the ability of avidin, streptavidin and chimeric avidin to specifically bind biotin at harsh conditions for the first time at the single molecule level. By looking into detail, they show clear differences in their functionality at the different environmental conditions studied. Avidin and its mutant chimeric avidin show a significant increased binding activity at very low pH values, whereas the structurally different streptavidin has its highest binding probability at pH 7, which gets somewhat linearly lowered when changing the pH in both directions. Thus, we suggest the use of streptavidin only at moderate pH conditions, whereas avidin seems to be perfectly suited for an extremely acidic environment. Chimeric avidin works fine over a broad range and shows comparable binding behavior at pH 1 and pH 11. It has to be mentioned that, in contrast to earlier studies, all given data represent the activity at (and not after exposure of) these harsh environmental conditions.

### 2.4. pH Induced Changes of the Bond Energy Landscape

A key advantage of single molecule experiments is the possibility to reveal the energy landscape of an interaction, especially if intermediate states appear in the unbinding process. Both avidin and streptavidin are known to have such states yielding in two (streptavidin) or three (avidin) energy barriers, visible in the loading rate dependence of the unbinding force. Each change of slopes in this plot corresponds to a different barrier according to Evans theory [10] and Bells model [36]. Not all regimes of loading rates are accessible by AFM, hence we show and discuss just one energy barrier here (*i.e.*, between 100 and 10,000 pN/s) since for both, lower and higher energy barrier insufficient range of the loading rate is accessible to perform accurate fitting. In dynamic force spectroscopy experiments, we compare avidin with its mutant chimeric avidin. For both proteins at pH7 , we report a barrier at x_β_ ≈ 4Å (*i.e.*, a distance of 4Å between the free-energy minimum and the maximum of the potential, measured in pulling direction), which coincides with the intermediate strength regime for avidin published by Merkel [10], De Paris [18], and Taninaka [23]. This barrier most likely constitutes an intermediate transition along the separation pathway, as the thermodynamically relevant transition state is generally assigned to the low force regime [37]. It should be mentioned that recent alternative theories allow a different interpretation of this behavior [27,38]. Thus, we also evaluated the data following the approach of Friddle and Noy [27,38]. Fitting the data of avidin-biotin at pH 7 according to Friddle and Noy resulted in a x_β_ of 4.27 Å, which is in good agreement, but both k_off_ (5.33 s^−1^) and the equilibrium force f_eq_ (21.06 pN) differed significantly from their evaluation of Merkels’ [10] avidin-biotin data (k_off_ = 0.75 s^−1^, f_eq_ = 6.1). Most probably the limited range of loading rate of our data does not allow accurate fitting of this model, thus we abandoned this fit for our data. The k_off_ values for the barrier corresponding to the investigated loading rate region (10^2^–10^4^ pN/s) vary significantly in previously published work [10,18,19,25,39], ranging from 0.08 s^−1^ [19] to 13.07 s^−1^ [25]. Our results for k_off_ of this avidin-biotin energy barrier at pH 7 is 2.37 ± 0.14 s^−1^ and thus in between the broad range of previously published results. It has to be mentioned that the off-rate corresponding to this barrier does not reflect the macroscopic dissociation kinetics, which are expected to be significantly slower. Nevertheless, our results show the influence of pH on this barrier of the bond energy landscape according to Evans in terms of kinetic off-rates (k_off_) and values for x_β_ shown in Table 1. The corresponding loading rate dependence of the unbinding force is shown in Figure 3c for avidin (left) and chimeric avidin (right). The lifetime of a transition state is given by the inverse off-rate (τ = 1/k_off_). Figure 3(b) shows the transition state lifetimes for both investigated proteins at different pH values. For avidin at neutral pH, this transition has a lifetime of τ = 0.42 s. In a more acidic environment, the lifetime is reduced to τ = 0.25 s at pH 3, then rises to τ = 0.33 s at pH 2 and τ = 0.48 s at pH 1. Most striking is the increased transition state lifetime at pH11 of τ = 1.14 s. Chimeric avidin shows a similar lifetime at pH 7 (τ = 0.36 s), varying the pH in both directions has also only minor effects, despite a drastic change at pH 1. In this extreme environment, the lifetime rises to τ = 1.32 s.

## 3. Experimental Section

### 3.1. Materials

#### 3.1.1. Chemicals

3-Aminopropyltriethoxysilane (APTES; Sigma Aldrich, Vienna, Austria) was distilled at low pressure and stored under argon in sealed crimp vials over silica gel (to avoid polymerisation) at −20 °C. MilliQ purified water (Millipore, Billerica, MA, USA) was used for all aqueous solutions. Triethylamine (TEA, Sigma Aldrich) was stored under argon in the dark to avoid amine oxidation. The heterobifunctional crosslinker Biotin-PEG-NHS was used as described previously [31]. Chloroform was purchased from J.T. Baker (Griesheim, Germany), and argon and N_2_ gas from Linde Gas GmbH (Stadl-Paura, Austria). Muscovite mica sheets were supplied by Christiane Gröpl Electron Microscopy (Tulln, Austria). Avidin and streptavidin were obtained from Sigma-Aldrich. Generation, purification and characterization of chimeric avidin were published previously [1,2].

#### 3.1.2. Cantilevers

For the experiments non-conductive silicon nitride MSCT tips were purchased from Bruker Corporation (St. Louis, MA, USA). For SMFS experiments the C-cantilever with a nominal spring constant of 0.01 N/m and for the scratch experiment the E-cantilever with a nominal spring constant of 0.1 N/m was used. The actual spring constant for the SMFS experiments was determined according to [40] using the thermal noise method. 

#### 3.1.3. Buffer

PBS 150 mM NaCl and 5 mM NaH_2_PO_4_ (pH adjusted with NaOH or HCl, respectively).

### 3.2. Methods

#### 3.2.1. AFM Tip Functionalization for SMFS Experiments

Silicon nitride AFM tips were functionalized with biotin by a well-established two-step procedure: (i) generation of amino groups on the tip surface and (ii) coupling of a heterobifunctional PEG linker via its NHS ester function to the amino-functionalized tips:

*Step 1.* In order to overcome the chemically inertness of the tips, the cleaned AFM tips were amino-functionalized. This was realized by the well-established APTES gas phase silanization method. Thus, the tips were prepared analogous to the optimized protocol [31]: AFM tips were washed three times for 5 min in chloroform and put into a 6 L desiccator, which is flooded with argon gas for half an hour to remove moisture and air. Afterwards APTES (60 µL) and TEA (20 µL) were separately pipetted into two small plastic trays and placed into the desiccator. Then, the AFM tips were positioned nearby on a clean inert surface (e.g., a Teflon^TM^ plate) and the desiccator was closed and rinsed with argon gas for 5 min. After two h incubation time, APTES and TEA vials were taken out from the desiccator. The desiccator was again flooded with argon for about 30 min, and the tips were left inside for 2 days in order to “cure” the APTES coating. For further use, the tips were stored under argon for less than seven days to avoid oxidation of the amine groups.

*Step 2.* The tethering of biotin on previously amino-functionalized AFM tips has been done in a simple one step reaction, since the ligand (biotin) is already part of the hetero-bifunctional crosslinker. For this reaction biotin-PEG(18)-NHS (1 mg, structure and synthesis described in [32]) was dissolved in chloroform (0.5 mL). The tips were transferred into this solution and TEA (30 µL) used as catalyst was carefully pipetted to it. After an incubation time of 2 h, the tips were rinsed three times for 5 min in chloroform to remove unbound linker and TEA, and dried in a gentle stream of nitrogen gas. Biotinylated tips were used immediately afterwards or stored under argon for less than one week.

#### 3.2.2. Sample Preparation

Avidin, chimeric avidin and streptavidin were covalently coupled to a mica sheet via a homo-bifunctional crosslinker in three step protocol: (i) amino-functionalization of mica sheets, (ii) attachment of the ethyleneglycol-bis(succinimidyl-succinate) (EGS) crosslinker molecule and (iii) coupling of the receptor molecule to the free end of the EGS-crosslinker:

*Step 1.* The modification of the mica sheets with aminofunctionalization reagents was performed as described in Section 3.2.1, Step 1 with the only difference that instead of washing, mica was mechanically cleaved before APTES coating.

*Step 2.* A flexible and short crosslinker for binding the receptor to the mica sheet was needed. Different strategies have been used so far for the specific and side-directed coupling of biomolecules to surfaces. In this study binding with EGS was performed. The protocol is described in [41] and works as follows: APTES amino-functionalized mica sheets were incubated for two h in a chloroform solution consisting of 1 mg/mL EGS and 100–200 µL TEA. Subsequently the EGS-APTES-coated micas were washed three times for 5 min in chloroform, dried in a gentle stream of nitrogen gas and used immediately afterwards.

*Step 3.* In the last step, the receptor molecule was incubated on the functionalized mica. In order to couple the proteins to the probe surface, 0.15 mg/mL receptor molecule (avidin/streptavidin/chimeric avidin) in phosphate buffered saline (PBS) was placed on the mica sheet for 2 h and finally washed 50 times with the same buffer. The protein functionalized sample can be stored for about 3 to 4 days in PBS at a temperature of 4 °C.

### 3.3. Experimental Approach

All measurements were performed on a commercial PicoSPM AFM (Agilent, Santa Clara, CA USA) equipped with a small scan-size scanner (x-y range: −2.9 to +2.9 µm; z-range: −1.4 to +1.4 µm) and carried out in aqueous liquid (PBS buffer with different pH values). Before each measurement, the fluid cell, sealing ring and tweezers were cleaned in the following order: (i) sodium dodecyl sulfate (SDS) for irreversible denaturation of protein debris, (ii) ultrapure water, (iii) isopropyl to remove SDS, and (iv) Millipore water again. To prevent contamination, the sample was washed before every measurement with the same pH value adjusted buffer (50 times) which was also used for measurements.

#### 3.3.1. Single Molecule Force Spectroscopy

For the investigation of the pH stability of the avidin and avidin-like proteins, 2000 force distance cycles were recorded for each pH value, each loading rate and each protein. Only at very low pulling velocities (≤100 nm/s) the number of FDCs was reduced to 500. All single molecule force spectroscopy measurements were performed with the functionalized MSCT-tips as described in 3.2.1. on the triangular cantilever C (nominal spring constant: 0.01 N/m). For the measurement, samples were placed in the pH value adjusted buffer. The loading rate was varied via the rate of piezo expansion (pulling speed in z-direction), which was adjusted by means of sweep duration time t [in s] and scan range s [in nm] for trace and retrace. To provide an insight into the loading rate dependence of the avidin-biotin complexes, pulling speeds from 50 nm/s to 3,000 nm/s were used. During each data set of 500–2,000 force distance cycles the tip position was changed each two hundredth curve by a few 100 nm to ensure that the binding probability was statistical independent from the lateral position on the sample surface. Before measurements at extreme pH values were performed, a reference measurement was done at a pulling speed of 400 nm/s at neutral pH (pH 7.00) in order to control the intactness of sample and biotinylated tip. The test for specificity, so called block experiment, was also done at a pulling speed of 400 nm/s and in neutral pH area (pH 7.00) for each of the three proteins. Therefore, the biotinylated tip was pre-blocked with streptavidin, so no more binding complexes could arise. Thus, all interactions (*i.e.*, downward bending of the cantilever in the retraction curve after detaching from the sample surface) visible after the block represent nonspecific binding.

For the spring constant determination the thermal noise method was applied [40]. To allow accurate results the spring constant determination of each tip used for force spectroscopy measurements was repeated five times. The binding probability of each dataset was calculated by the number of FDCs showing a ligand receptor interaction (*i.e.*, a downwards bending in the retraction part) divided by the total number of FDCs collected at these conditions. All experiments were repeated 3–4 times (resulting in 2,000–8,000 FDCs per pH and pulling velocity) and the number of dataset repeats is given after the binding probability in the result section as n. Distributions of unbinding forces were obtained by constructing empirical probability density functions from the unbinding force measurements. There the maximum in the PDF reflects the most probable unbinding force, whereas the broadness corresponds to the standard deviation [33]. The loading rate of every curve was calculated by multiplying the applied pulling velocity with the effective spring constant (*i.e.*, the slope just before the unbinding event). The kinetic off-rate constant k_off_ and the distance of the energy barrier form the free equilibrium position x_β_ were determined using a maximum likelihood approach as described in [42] and fitting the data according to the single-barrier model [9]. There the rupture force F* is given as function of the loading rate with F* = f_β_ ln(r/(k_off_ f_β_)), where f_β_ is the ratio of the thermal energy k_B_T and x_β_. For avidin and chimeric avidin only unbinding events of a loading rate between 100–10,000 pN/s were used for calculating the parameters of the energy landscape (k_off_ = 1/ τ, x_β_).

#### 3.3.2. AFM Imaging

In order to prove the quality of the sample preparation (formation of a dense protein monolayer), a scratch image was performed. In step 1, the sample was imaged in contact mode, where the tip was moved laterally over the surface at constant force. Afterwards in step 2, the scan range was reduced to a smaller area and a higher force (~500 pN) was applied to the protein monolayer during scanning. Thereby the bound molecules were scratched away and bare mica was exposed. In the last step, the scan range was increased again and the surrounding of the scratched area was imaged. The topography images were displayed after leveling by mean plane subtraction and scanning line correction (Gwyddion 2.9 and 2.34). The height difference between layer and the bare mica were measured using the extract profiles tool in Gwyddion.

## 4. Conclusions

In conclusion, we could demonstrate the extraordinary high stability of both avidin and chimeric avidin towards biotin at harsh pH conditions. The binding probability, reflecting the kinetic of complex formation, was investigated for avidin, chimeric avidin and streptavidin under different harsh pH conditions ranging from pH 1 to 12.75. Within the pH range of 2–11 neither avidin nor chimeric avidin show any noteworthy decrease of complex formation probability, and surprisingly the binding probability of avidin-biotin even increases significantly at pH 1. In contrast, streptavidin has a maximum at physiological pH and shows a significant decrease at lowered and increased pH values. For the investigation of the unbinding behavior we limited our research to avidin and chimeric avidin, since it is known that these proteins have a different energy barrier in their energy landscape at the loading rate region addressable with the AFM compared to streptavidin. The τ values, reflecting the lifetime of the relevant state in the energy landscape of the complex with the barrier at x_β_, are similar for both, avidin and chimeric avidin but show a significant increased complex stability at pH 1 for chimeric avidin and at pH 11 for avidin. Thus, in case of applications based on the recognition of avidin-biotin near pH 1 where the on-kinetic (complex formation process) is limiting avidin shows a better performance, whereas in applications which are limited by the dissociation process chimeric avidin represents the better choice. At pH 11, avidin is preferable and at all pH values in between both, avidin and its mutant chimeric avidin, behave similarly. This understanding allows realizing applications based on avidin/chimeric avidin at extreme pH values.
